# Are there moral differences between maternal spindle transfer and pronuclear transfer?

**DOI:** 10.1007/s11019-017-9772-3

**Published:** 2017-04-20

**Authors:** César Palacios-González

**Affiliations:** Centre of Medical Law and Ethics, The Dickson Poon School of Law, King’s College London, Strand, London, WC2R 2LS UK

**Keywords:** Mitochondrial replacement techniques, Mitochondrial replacement therapy, Mitochondrial donation, Maternal spindle transfer, Pronuclear transfer, Tri-parenthood, Three parent babies, Three parent IVF

## Abstract

This paper examines whether there are moral differences between the mitochondrial replacement techniques that have been recently developed in order to help women afflicted by mitochondrial DNA diseases to have genetically related children absent such conditions: maternal spindle transfer (MST) and pronuclear transfer (PNT). Firstly, it examines whether there is a moral difference between MST and PNT in terms of the divide between somatic interventions and germline interventions. Secondly, it considers whether PNT and MST are morally distinct under a therapy/creation optic. Finally, it investigates whether there is a moral difference between MST and PNT from a human embryo destruction point of view. I conclude, contra recent arguments, that regarding the first two points there is no moral differences between PNT and MST; and that regarding the third one MST is morally preferable to PNT, but only if we hold a gradualist account of the moral value of human embryos where zygotes have slight moral value.

## Introduction

Mitochondrial DNA diseases (mtDNA diseases) occur when problems within the genes of the mitochondria prevent them from producing the levels of energy cells need to work properly. They are a group of neuromuscular diseases that can have mild to devastating effects. They cause, for example, heart and major organ failure, dementia, stroke, blindness, deafness, infant encephalopathy, and premature death (Department of Health 2014). Mitochondria are inherited via the maternal line (men affected by such diseases do not, generally, transmit them to future generations^1^); and pathological mutations in the mitochondrial DNA can be present either in all mitochondria, referred to as ‘homoplasmy’, or only in some mitochondria, known as ‘heteroplasmy’.

Recently two mitochondrial replacement techniques^2^ (MRTs) have been developed in order to help women with mtDNA diseases to have genetically related children absent such conditions: maternal spindle transfer (MST) and pronuclear transfer (PNT). In MST assisted reproductive techniques are used to obtain eggs from the intending mother and a healthy donor. The chromosomes from the donor’s oocyte and the intending mother’s oocyte are then extracted. Whilst the donor’s chromosomes and the intending mother’s enucleated oocyte are discarded, the intending mother’s chromosomes are transferred to the now enucleated donor’s oocyte.^3^ Afterwards, the reconstructed oocyte is fertilised in vitro and then transferred to the intending mother or a surrogate (Tachibana et al. 2009; Nuffield Council on Bioethics 2012).

In PNT two zygotes are created in vitro. One of them is created with the intending parents’ sperm and oocyte (or a sperm from a donor), and the other one with a donated oocyte and the father’s (or donor’s) sperm. After fertilisation, and during the first 24 h, the maternal and paternal pronuclei are removed from both zygotes. The enucleated cell that was produced with the intending mother’s oocyte and the pronuclei that were contained in the cell produced with the donor’s oocyte are discarded. Subsequently, the intending parents’ (or donor’s and intending mother’s) pronuclei are transferred to the enucleated cell produced with the donor’s oocyte. The reconstructed zygote is then transferred to the intending mother or a surrogate (Craven et al. 2010; Nuffield Council on Bioethics 2012). In both techniques the donor’s healthy mitochondria will be passed down via the maternal line to subsequent generations, if everything goes as expected.

Until now most of the academic literature on the ethics of MRTs has focused on the question of whether *both techniques* are morally permissible.^4^ In this paper I depart from this path, and instead examine whether there are moral differences *between* PNT and MST. The paper focuses on this issue because when we have to choose between two options, other things being equal, we should prefer that which is morally superior. Additionally, mapping the moral differences between these techniques could help in easing their *explicit* regulation both in traditionally liberal and conservative jurisdictions. It can do so in that it would become clear how other existing laws and regulations would apply to these techniques, and also, which laws and regulations should be modified in order for both MRTs, or one of them, to be made explicitly legal.

In the following sections I examine three possible reasons for why MST and PNT are morally distinct from each other. First, I examine whether there is a moral difference between them in terms of the divide between somatic interventions and germline interventions. Second, I consider whether they morally differ under a therapy/creation optic. Finally, I investigate whether they morally differ from a human embryo destruction point of view. I conclude, contra recent arguments, that regarding the first two points there is no moral differences between PNT and MST; and that regarding the third one MST is morally preferable to PNT, but only if we hold a gradualist account of the moral value of human embryos where zygotes have slight moral value.

## Germline/somatic interventions

One way in which we could try to elucidate a moral difference between PNT and MST is by investigating whether one constitutes a germline intervention whereas the other is a somatic one. This is particularly relevant since both MRTs have been constantly criticised for being germline modifying techniques. For example, Marcy Darnovsky has asserted:

Mitochondrial-replacement procedures *would constitute germline modification* [emphasis added]. Were the United Kingdom to grant a regulatory go-ahead, it would unilaterally cross a legal and ethical line on this issue that has been observed by the entire international community. This consensus holds that genetic-engineering tools may be applied, with appropriate care and safeguards, to treat an individual’s medical condition, but should not be used to modify gametes or early embryos and so manipulate the characteristics of future children (Darnovsky 2013).

The difference, *in biological terms*, between these kinds of interventions is that whilst somatic interventions *are not inheritable*, germline interventions *are inheritable*. According to the *mainstream ethical position* somatic modifications are morally preferable to germline ones.

Essentially all observers have stated that they believe that it would be ethical to insert genetic material into a human being for the sole purpose of medically correcting a severe genetic defect in that patient, in other words, somatic cell gene therapy. Attempts to correct a patient’s reproductive cells (i.e., germ line gene therapy) or to alter or improve a ‘normal’ person by gene manipulation (i.e., enhancement or eugenic genetic engineering) are controversial areas (Anderson 1985, pp. 277–778).

Germline interventions are morally *not to be preferred* because their effects *will be* passed on (if the affected individual reproduces and other conditions are met) to future generations. This means that if the intervention causes an unwanted effect, for example causes the individual to be in extreme pain, then this effect could be passed on—this argument against germline interventions is precautionary in nature. In its recent report on MRTs the US Institute of Medicine gives weight to this intergenerational worry:

Concerns about the risk of heritable change and the effects on future generations *are valid and important* [emphasis added], and both restrictions on the application of MRT and the collection of information about its effects would be crucial aspects of acceptable policies that would have to be in place for MRT investigations to proceed (Institute of Medicine of the National Academies 2016, p. 94).

If we accept, *at face value*, the position that there is a moral difference between somatic and germline interventions then we can investigate whether there is a moral difference between MST and PNT in this regard. There is, however, no moral difference between MST and PNT when examined from the somatic/germline optic. This is so as both can be instances of *germline interventions* and of *somatic interventions* (Palacios-González 2016, p. 47–48; Institute of Medicine of the National Academies 2016, p. 89; Bredenoord et al. 2011, p. 100).

If we choose for *female* embryos, through sperm sorting or preimplantation genetic diagnosis, *both* MST and PNT would be instances of *germline intervention*, since the modification would be passed on to future generations given that mitochondria are maternally inherited. If, alternatively, we choose for *male* embryos then both techniques would be instances of *somatic interventions*, since such modifications would not be passed on to future generations as mitochondria are maternally inherited. Therefore, we can confidently assert that there is no moral difference between PNT and MST from a somatic/germline optic.

## Therapy/creation acts

A second way in which we might attempt to identify a moral difference between MST and PNT is by investigating whether one of them is a *therapeutic act* whereas the other is, what might be termed, a *creation-related act*. A therapeutic act is one carried out upon an existing (or merely determinate) individual in a medical context, and we can say that as a consequence of it someone can be better off or worse off. By creation-related act we should understand an act does not affect the interests or prospects of a particular (existing or future) person but instead determines (partially or totally) *who it is* that will come to exist. Regarding identity, I am here following Parfit’s origin view: “(…) each person has this distinctive necessary property: that of having grown from the *particular pair of cells* [emphasis added] from which this person in fact grew” (Parfit 1984, note 11, p. 352).

When having to choose between such acts in situations of scarce medical resources, other things being equal, therapeutic acts are considered to be morally superior. This is so given that they make someone *better off*, whereas creation-related acts *only bring someone into existence* and this is a neutral action [with the exception of wrongful life cases (Feinberg 1986)] under a person-affecting account of morality. In a recent paper Anthony Wrigley et al. (2015) have argued that PNT is a *therapeutic intervention* (a ‘pre-emptive cure’) whereas MST is a kind of *selective reproduction*: “PNT, it is argued, is a form of therapy based on embryo modification while MST is, instead, an instance of selective reproduction” (Wrigley et al. 2015, p. 631). These authors conclude that *there are moral reasons* for offering PNT over MST (supposing that both are equally safe, effective and cost-effective): “Thus there is a strong *prima facie* harm-avoidance rationale for offering PNT to prospective parents, and for those parents to accept it; one that is not present in the case of MST” (Wrigley et al. 2015, p. 636).

The way in which these authors defend their claim is by examining the mechanics of PNT and MST. When *‘the process of MST’* (which entails the enucleation, transfer and reconstitution actions) is carried out *there is no individual* in relation to which the technique is applied. There is no individual because the process of MST occurs on *unfertilized eggs*, and hence we are lacking half of the chromosomes for having one (i.e. a sperm). Even more so, when we have the MST-egg (the MST-egg is the end-result of ‘the process of MST’) ready for fertilisation we still do not know the identity of the future individual since which sperm will fertilise the MST-egg is contingent on a multitude of factors, from when the sample is collected to how the sample is handled. The only case where this *does not attain* is where we have preselected a particular pair of gametes before MST is considered as a reproductive option.^5^ In conclusion, in ordinary cases the *process of MST does not treat anyone*, and we should regard using an MST-egg for a reproductive endeavour as a creation-related act.^6^

Wrigley et al. maintain, on the other hand, that PNT can be a form of therapy for a particular subject. This is so because PNT happens when there is already an individual:

With PNT, however, the intervention happens after fertilization, the gametes used are unaffected, and so the non-identity problem does not arise. This means that PNT is capable of benefiting or harming any child created in a straightforward ‘harm-to-interests’ sense.(Wrigley et al. 2015, p. 635)

It must be said that Wrigley et al.’s position regarding *‘the process of PNT’* (which also entails the enucleation, transfer and reconstitution actions) is *correct*, in that it does not causally affect the identity of the intending parents’ would be enucleated zygote.^7^ It does not do so because a particular sperm and egg had already fused prior to the process of PNT taking place. Therefore, the intending parents’ *unmodified-zygote* can in fact be made better off or worse off by the process of PNT, supposing it is the case that ‘numerical identity follows the nuclear genome’.^8^ In other words, *the process of PNT can be regarded as therapeutic* and thus the Non-Identity Claim *is not* satisfied by it. The Non-Identity Claim:

When we use technique x this causes a (numerically) different person to be born, i.e. someone other than the person who would have been born if we had not used technique x.

Now, an important caveat regarding Wrigley et al.’s examination of PNT should be acknowledged, they do not take into consideration the *‘clinical decision to employ PNT’*. This is important because the clinical decision to employ PNT in fact alters the timing of conception and thus who will be brought into existence.^9^

For all those reproductive scenarios where PNT is the chosen course of action and where the specific gametes that will fuse *have not been* preselected prior to the decision to employ the technique, the clinical decision to employ PNT *causally affects which gametes will fuse*. This is so because after opting for PNT the woman would have to go through assisted reproductive techniques to obtain her eggs (if she does not have some stored) while her partner (or a donor) would have to provide a sperm sample.^10^ This means that the gametes that will fuse in order for the process of PNT to happen *would most certainly not have fused* in the first place if PNT had not been chosen as the course of action.^11^ At this point we can conclude that both *the clinical decision* to employ PNT and *the clinical decision* to employ MST alter the timing of conception and thus both are creation-related acts. Thus, from a therapy/creation optic there is no moral difference between *the clinical decision* to employ PNT and *the clinical decision* to employ MST. Both decisions bring someone into existence that otherwise would not have existed.

Now, this finding shows, contra Wrigley et al.’s conclusion, that there is no harm-avoidance rationale *for offering as a reproductive option* PNT over MST to women with mtDNA diseases who want to have genetically related children.^12^ This is so because there is no individual *at that point* that would benefit from PNT, just as as there is no one *at that point* that would benefit from MST.^13^ The clinical decision to employ MST or PNT differs from a truly therapeutic decision, for example the resolution to vaccinate a young adult whose existence is no way related to the vaccination programme.^14^

### Harm and the medical practice of PNT and MST

Once we have established the former let’s look at the *medical practice* of both MRTs—which entails the clinical decision to employ MST (or PNT), the process of MST (or PNT) and the use of an MST-egg (or PNT-zygote). The clinical decision to employ MST is identity affecting and the use of an MST-egg is also identity affecting. This means that the *medical practice of* MST is, so to speak, ‘doubly’ identity affecting. Therefore, in all those cases where no pair of gametes were preselected previous to the decision to employ MST (which are most, if not all of them) the medical practice of MST cannot harm the child produced, since it does not make the child worse off than she would otherwise have been. It does not make her worse off than she would otherwise have been because the only options available are to be conceived in this way or to not exist at all.

For PNT things are different, the clinical decision to employ PNT is identity affecting but the process of PNT itself is not identity affecting. Thus, the *medical practice of* PNT is not ‘doubly’ identity affecting but ‘single’ identity affecting. This entails that even when we do not have harm-avoidance reasons *for offering as a reproductive option* PNT over MST, once we have decided for PNT and we have produced an embryo, the process of PNT can make an individual better off or worse off. Why? Because the decision to employ X can bring Y into existence (which does not make Y better off or worse off), but the use of X on Y, once Y exists, can make Y better off or worse off than she would otherwise have been. For example:

Imagine that woman K is homoplasmic for a mild mtDNA disease and chooses to employ PNT. The clinical decision to employ PNT affects the timing of conception and brings about embryo L. If the decision to carry out PNT had not been made an alternative embryo, embryo M, would have existed. Afterwards the process of PNT is carried out on embryo L. In this scenario the process of PNT can make L better off or worse off than she would otherwise have been. For example, if the procedure was completely safe and effective it would make L better off than she would have been if the intervention did not occur, since absent the intervention L would have a mtDNA disease. Alternatively, if during the process of PNT there was enough mutant mtDNA carryover so for the disease to manifest, *in addition to the procedure causing lifelong extreme pain*, then the process of PNT would make L worse off than she would have been if the intervention did not occur, since absent the intervention L would only suffer from a mild mtDNA disease.^15^^,^ 16

Even when most PNT *real life cases* will be like the ones just presented, it is possible to construct a case where a PNT-zygote *would not have been made worse off* by virtue of the process of PNT, even if in the end the PNT-conceived individual suffers more than if she had only had the mtDNA disease.^17^ *This is not to say that the process of MST and the process of PNT are equivalent with respect to non-identity concerns*. What I am asserting is that because a counterfactual account of harm examines ‘how things would otherwise have been’, there are certain instances where the alternative would be worse than being subject to PNT. Imagine the following: woman N is homoplasmic for a mtDNA disease and she irrevocably decides that her embryos will only be produced in vitro and undergo PNT or that she will destroy them. Because she does not want anyone else to intervene (and possibly halt her plans), she has automatized all the procedure so she is capable of doing it all by herself. As we know by now, the clinical decision to employ PNT affects the timing of conception and, let’s suppose, embryo O is produced. Now, the process of PNT in this particular instance does not make O worse off than she would otherwise have been, even if its effects are worse than the mtDNA disease.^18^ O is not made worse off by the process of PNT because the only other available options for O is to be destroyed. In other words, in this case the process of PNT does not make O worse off than O would have been if it did not occur, because if it had not occurred then O would have been destroyed and this would have been worse for O (Boonin 2014, p. 62).

Setting aside the previous caveat regarding a counterfactual account of harm and PNT, let’s conclude this section by restating that from a creation/therapy optic *the clinical decision* to employ PNT or MST brings someone into existence that otherwise would not have existed. Thus there is no harm-avoidance rationale *for offering as a reproductive option* PNT over MST to a woman with a mtDNA disease that wants to have a child that is genetically related to her.

### PNT and MST and the Organism View

All the previous discussion on numerical identity is grounded on the account that ‘numerical identity follows the nuclear genome’. In a recent paper Liao (2017) has diverted from this position and has defended, following the Organism View, that eggs are *essentially* cells and that zygotes are *essentially* organisms. This means that eggs and zygotes: begin to exist when their capacity to regulate and coordinate the various life processes is there; persists as long as there is ‘organismic (or cellular) continuity’, which is the continuing ability to regulate and coordinate the various life processes; and ceases to exist when the capacity to regulate and coordinate the various life processes ceases to be (Liao 2017, p. 24). Human eggs’ and zygotes’ ‘organismic (or cellular) continuity’ depends, among other things, on the correct interaction between the nucleus and the mitochondria. It does so given that *both the mitochondria and the nucleus are essential* to control the various life processes: there are life processes in the mitochondria that the nucleus does not (at least have full) control, and there are life processes in the nucleus that the mitochondria does not (at least have full) control (Liao 2017, p. 23).

If the above is correct then *both the process of PNT and MST alter numerical identity*. They do so since when we carry them out the enucleation procedure *disrupts* the zygote’s (or egg’s) organismic (or cellular) continuity, and thus when we transfer the couple’s nuclear DNA (or intending mother’s nuclear DNA) to the enucleated cell we create a new being with a new organismic (or cellular) continuity. Under this interpretation of the Organism View the *clinical decision* to employ PNT or MST are identity affecting, per the same reasons presented in the previous sections, and both the process of PNT and MST are identity affecting. Therefore, the process of PNT cannot harm those created through it since the only options available are to be created in this way or to not exist at all, contrary to what it is the case in ‘numerical identity follows the nuclear genome’.

## Embryo destruction

A third way in which we could try to identify a moral difference between PNT and MST is by examining both techniques from a human embryo destruction optic. When we focus on this issue a clear difference between both MRTs becomes evident: the medical practice of PNT *requires the destruction of an embryo*, whereas the MST one only requires the destruction of an *unfertilized egg*.^19^ This difference has prompted some to argue that we should mainly focus on MST, while forgoing PNT. For example, Lucía Goméz-Tatay et al. have claimed:

We also believe that research efforts should not be divided between MST and PNT, since the latter, besides not presenting any advantage in terms of safety and efficacy, presents more ethical difficulties, insurmountable for many, that must be taken into account in the context of a pluralistic society (Gómez-Tatay et al. 2016, p. 16).

It is evident that the answer to the question of whether there is a moral difference between MST and PNT, from a human embryo destruction point of view, inheres in questions about the morality of the intentional destruction of human embryos. Even though this is *in a strict sense* the case, I do not have the scope here to defend a particular position on this topic. Rather, I will examine whether there are moral differences between MST and PNT from the point of view of the three most common positions on the moral value of human embryos: the liberal position, the conservative position and the gradualist position.

### Liberal position

Those who defend a liberal position find *no moral difference between PNT and MST*. Both MRTs *are morally on a par* because human embryos are not inherently valuable. They argue that human embryos do not posses intrinsic moral value because they are not persons (Warren 2002), they do not posses a future like ours (Sinnott-Armstrong 1999), and their potentiality (Harris 2006) and the fact that they belong to the human species (Singer 2011) does not bestow upon them moral value. Thus, the destruction of early human embryos in order to carry out PNT is as morally inconsequential as the destruction of human eggs for carrying out MST.

### Conservative position

The conservative position, on the other hand, holds that the human embryo possesses high intrinsic moral value (Finnis 1973; Stone 1987). On this view, the intentional destruction of human embryos is tantamount to the killing of innocent adult human persons.^20^ Therefore, a woman, or couple, who holds this view should regard PNT as morally impermissible because it necessitates the intentional destruction of a human embryo for the benefit of another one. In PNT the woman, or couple, knowingly and intentionally instructs another person to create and then kill an early human embryo.^21^ This is equivalent to instructing and authorizing the killing of an innocent teenager in order to harvest his heart to help another teenager. Furthermore, even if the woman, or couple, does not destroy the embryo herself she is *directly encouraging it through agency* and thus she is completely morally responsible for its destruction (Green 2002).

Now, given that in the *medical practice* MST does not *inherently* require the destruction of human embryos it seems that conservatives would be able to morally resort to it.^22^ Therefore, we could be tempted to conclude that in terms of embryo destruction there is an insurmountable moral difference between MST and PNT. Even though the former seems reasonable, in what follows I will show that *when we factor in all the present variables relating to* MST it becomes clear that it is out of the moral reach of conservatives.

*At this point in time* MST requires the destruction of human embryos because it is in its development phase. In order to investigate whether the technique works in humans,^23^ and whether embryos develop normally *after MST*, scientists need to create human embryos for the sole purpose of researching onto them and afterwards those embryos will be *discarded*. This means that *before MST moves into assisted reproduction centres* many human embryos will be intentionally destroyed during research. As the US Institute of Medicine’s notes:

Any preclinical data required by regulators for consideration in advance of first-in-human investigations [of MRTs] could increase the numbers of embryos created, many of which would likely not be transferred for implantation.(Institute of Medicine of the National Academies 2016, p. 104)

Furthermore, intentional embryo destruction in the MST context is not limited to the initial developmental phase, but would also occur if and when major changes are introduced in the way in which the technique is carried out. If there were significant improvements or variations to the technique then embryos would also be created and destroyed while researching the safety and efficacy of the modified MST technique.

Conservatives arguing for only-MST, on embryo destruction grounds, cannot actually endorse this technique. First, even if no agency relationship exists between the woman, or couple, and the destruction of human embryos, they would be *directly encouraging their destruction through the acceptance of benefit* (Green 2002, p. 549). In this case the woman, or couple, benefits from what she considers as a truly morally pernicious deed and her acceptance of this benefit directly encourages those destroying embryos to keep on doing so, even if her role in the overall encouragement is small. Additionally, if no calls for punishment and criticising of the scientists working on MRTs happen then they would most probably receive academic rewards (both material and psychological) for their work and the encouragement to keep on doing research that includes embryo destruction. If conservatives do not want to directly encourage the destruction of human embryos through the acceptance of benefit then they have to reject MST.

The second reason why conservatives cannot endorse MST is because they would be *indirectly encouraging the destruction of human embryos through the legitimization of such practice* (Green 2002, p. 550). In this case we are not interested in how the benefits particular scientists could obtain would directly encourage them to do similar research. Rather, what we are interested in is that accepting the benefits of MST *socially legitimises* scientific practices that intentionally destroy human embryos. If conservatives are willing to use the results of research that requires embryo destruction then what they are effectively saying is: society “may use scientific information that has been created by wicked research on human subjects so long as the information can benefit” (Green 2002, p. 550) woman with mtDNA diseases who want to have genetically related children. Given that conservatives do not want to socially legitimise embryo destruction practices then they have to reject MST.

Additionally, someone could further argue that a typical assisted reproduction cycle that included MST would also end up in the intentional destruction of human embryos. Because during assisted reproduction cycles many embryos are created and only some of those deemed healthy are transferred, whilst the other ones are cryopreserved or destroyed. The problem with this third reason is that this is not *an essential feature* of MST. Those supporting only-MST could advocate that only two or three embryos were created per cycle, for example, and that all of them were always transferred to the intending mother or a surrogate.

At this point we can conclude that in practice at the present moment, for conservatives, there is no moral difference between MST and PNT in terms of the intentional destruction of human embryos. PNT requires the intentional destruction of human embryos; and MST indirectly encourages the destruction of human embryos through the legitimization of such practice, and directly encourages the destruction of embryos through the acceptance of benefit. If conservatives do hold that human embryos possess high moral value then they cannot overlook these features of MST research.

### Gradualist position

The gradualist position maintains that human embryos’ (and then foetuses’) moral value gradually increases as they develop through pregnancy (Scott 2007; Dworkin 1993). Thus “increasingly strong reasons are needed to try to justify the loss of fetal life” (Scott 2007, p. 2) as pregnancy elapses. Even when this is the case the gradualist position also holds that “the newly conceived embryo has no moral value (or extremely little)” (Kaczor 2010, p. 88). If we accept that the human early embryo does not possess moral value then both PNT and MST are morally on a par in terms of embryo destruction, for the same reasons presented when discussing the liberal position.

If we, on the other hand, accept that human embryos possess *very little moral value*, and that it is morally permissible to destroy them for allowing a woman or couple to have a genetically related child, then we have to accept that MST is (slightly) morally preferable to PNT. This is because the total number of embryos destroyed in PNT would be higher than that in MST, when considering both clinical and research practices. Let’s remember that each PNT process requires the intentional destruction of embryos, whereas this is not the case in MST. We can conclude that for a gradualist who ascribes little moral value to human embryos the development and use of MST is morally preferable, since it minimises the destruction of something that is minimally morally valuable.

## Conclusion

In this paper I have examined whether there are moral differences between MST and PNT on three grounds: somatic/germline, therapy/creation, and embryo destruction. Even though it seemed plausible to find moral differences between both MRTs on these three grounds, the reality is that the only moral difference between them is on embryo destruction grounds, if we hold a view that confers minimal moral value to human embryos. For such gradualists, MST is morally preferable to PNT, because the *overall* destruction of something with moral value is minimized. A last word: even if my findings do not *completely* settle the question of whether there are significant moral differences between these techniques, they at least have shown that the first two grounds cannot be used in order to *radically* morally differentiate between PNT and MST.

## Footnotes

1 Only one case of mitochondrial DNA inheritance via the paternal line has been documented in the academic literature (Schwartz and Vissing 2002).

2 Even when the name ‘mitochondrial replacement techniques’ is controversial I use it because it has secured a foothold within the academic debate, see Palacios-González (2016, p. 40). The name is controversial because the techniques transfer *nuclear DNA* and thus a better term would be ‘nuclear replacement techniques’. Paying attention to this fact is important in order no to mischaracterise the techniques as intentionally replacing mitochondria, as Tina Rulli does: “MRTs *replace the defective mitochondria* [emphasis added] from the intended genetic mother’s egg with the healthy mitochondria from a donor woman’s egg” (Rulli 2016b, p. 2). Newson and Wrigley (2017) have recently proposed and defended the term ‘mitochondrial targeting techniques’.

3 During the chromosome transfer there can be unintentional carryover of pathological mitochondria. The mtDNA disease could manifest if the pathological mitochondria carryover is substantive (Yamada et al. 2016).

4 For a review of the issues that have been explored in the literature see: ‘Ethics of mitochondrial replacement techniques: a Habermasian perspective’ (Palacios-González 2017a).

5 Although this situation is feasible it is not the standard practice in reproductive scenarios.

6 The case just described is *structurally symmetric* to other very well known non-identity cases, for example ‘the 14 years old girl’ case described by Parfit in his ‘Reasons and Persons’ (Parfit 1984, p. 358).

7 For an overview of the non-identity problem see Roberts (2015). For an exposition of why there is no such thing as a non-identity problem see Boonin (2008, 2014).

8 Even when much of the MRTs literature presupposes that ‘numerical identity follows the nuclear genome’ this position has not been argued for.

9 This fact about PNT was first identified by Palacios-González (2016, note. 12 p. 46, 2017b) and Rulli (2016a).

10 This is also true of MST: the clinical decision to employ MST affects the timing of conception.

11 In actuality, there is no metaphysical impossibility for the same gametes to fuse, but the probability of that being the case is negligible.

12 This conclusion also follows, in most cases, if we hold a weak origin view, as shown by Rulli (2016b). According to the weak origin view the numerical identity of the individual persists as long as it is the same egg that that is fertilised. In other words, a change in sperm is not identify-affecting. This conclusion also follows because the clinical decision to employ PNT affects the timing of conception in a matter of months, and not only to days, given the time needed for the ovarian stimulation to take place.

13 In a paper that was published while this paper was under review, Tina Rulli has also pointed out that the clinical decision to employ PNT alters the timing of conception and thus it should not be characterised as therapeutic. One issue with Rulli’s paper is that throughout it she fails to properly specify when she is talking about ‘the clinical decision to employ PNT’ and when she is talking about ‘the process of PNT’. For example: “But PNT does not treat an existing person or a person who is on its way into existence. Rather the child will exist just because PNT is selected to be used in her creation” (Rulli 2016b, p. 5).

14 I am aware that most cases can be modified to produce non-identity scenarios.

15 Two real life cases could be as follow: L’s parents could have a change of heart in the last second about PNT, or there could be confusion in the clinic and embryo L could be transferred to the intending mother without the process of PNT occurring. Supposing the process of PNT is safe and effective both these scenarios would leave L worse off than she would otherwise have been, since the alternative in both cases is for the process of PNT to ensue and L not to have a mtDNA disease.

16 In her treatment of PNT Rulli misses this important point, for she only focuses on the possible benefits that the process of PNT could confer: “There is a trivial sense in which the child produced by PNT is benefited—that is, she is not harmed as she could have been had PNT not been used” (Rulli 2016b, p. 4).

17 Supposing that the individual’s life is still not a wrongful one.

18 Assuming that her life is still not a wrongful one.

19 Under a ‘numerical identity follows the nuclear genome’ account only one embryo is destroyed, under the organism view account two embryos are destroyed.

20 Here I am using person in the Lockean sense: a person is “a thinking intelligent being, that has reason and reflection, and can consider itself as itself, the same thinking thing in different times and places” (Locke 1975, p. 335).

21 Here I am drawing from Green’s (2002) “Benefiting From ‘Evil’”.

22 It is important to emphasise that I am assuming that both techniques are equally safe and effective.

23 There has been proof of principle that a human live birth is possible after MST and PNT (Zhang et al. 2016; Coghlan 2017).
